# First Imported Case of Chikungunya Virus Infection in a Travelling Canadian Returning from the Caribbean

**DOI:** 10.1155/2016/2980297

**Published:** 2016-02-24

**Authors:** Christian Therrien, Guillaume Jourdan, Kimberly Holloway, Cécile Tremblay, Michael A. Drebot

**Affiliations:** ^1^Laboratoire de Santé Publique du Québec, Institut National de Santé Publique du Québec, 20045 chemin Sainte-Marie, Sainte-Anne-de-Bellevue, QC, Canada H9X 3R5; ^2^Hôpital de Chicoutimi, Centre de Santé et de Services Sociaux de Chicoutimi, Chicoutimi, QC, Canada; ^3^National Microbiology Laboratory, Public Health Agency of Canada, Winnipeg, MB, Canada

## Abstract

This is the first Canadian case of Chikungunya virus (CHIKV) infection reported in a traveller returning from the Caribbean. Following multiple mosquito bites in Martinique Island in January 2014, the patient presented with high fever, headaches, arthralgia on both hands and feet, and a rash on the trunk upon his return to Canada. Initial serological testing for dengue virus infection was negative. Support therapy with nonsteroidal anti-inflammatory drugs was administered. The symptoms gradually improved 4 weeks after onset with residual arthralgia and morning joint stiffness. This clinical feature prompted the clinician to request CHIKV virus serology which was found to be positive for the presence of IgM and neutralizing antibodies. In 2014, over four hundred confirmed CHIKV infection cases were diagnosed in Canadian travellers returning from the Caribbean and Central America. Clinical suspicion of CHIKV or dengue virus infections should be considered in febrile patients with arthralgia returning from the recently CHIKV endemic countries of the Americas.

## 1. Summary

The present report is the first published case of a Canadian patient acquiring a Chikungunya virus (CHIKV) infection after travelling to a newly endemic country in the Americas. The CHIKV was introduced in the island of Saint-Martin in December 2013 and spread rapidly to neighboring countries in the Caribbean, South, Central, and North America (Florida) through local transmission cycles. There is no treatment to cure the disease; supportive treatment with nonsteroidal anti-inflammatory drugs is available to relieve symptoms. The clinical picture of CHIKV and dengue virus infection shares several similar signs and symptoms. Therefore, a prompt request of laboratory tests for dengue and Chikungunya viruses in travellers returning from America endemic regions is recommended to confirm an arboviral disease and to adjust the treatment options. Other pathogens which mimic Chikungunya infection such as Zika virus,* Leptospira *spp.,* Plasmodium* spp., and other viruses should also be considered in the differential diagnosis.

## 2. Case Presentation

The patient is a 57-year-old Caucasian male with no past relevant medical history. He travelled to Martinique from mid-January to February 2, 2014, where he stayed in a villa, in the suburb of the city, close to the mountains. The villa was well maintained, with no evidence of mould. The patient recalled multiple mosquito bites, mostly during the first few days of his stay. No other particular infectious exposition was noted during his activities, and he did not experience episodes of fever, gastrointestinal or respiratory illness during his trip.

About three days upon his return to the province of Québec, Canada, he presented with a viral syndrome including myalgia, reduced general condition, fever, and increasingly progressive headaches over two to three days. On February 7, he sought medical advice for arthralgia in both hands and feet, joint swelling/synovitis, and a nonpetechial rash on the thorax. The workup was negative for renal and liver anomalies but did reveal leucopenia (3.1 × 10^9^ cells/L) and a discrete neutropenia and lymphopenia (1.6 and 0.7 × 10^9^ cells/L, resp.). No eosinophilia was noted, and the platelets levels remained normal. Further analysis revealed a negative urine culture and a normal sedimentation rate.

In order to confirm a putative infection with dengue virus, serological tests for dengue infection were ordered on paired sera. The acute serum was collected February 7 and the convalescent serum collected two weeks later. Both sera were negative for the presence of anti-dengue IgM and IgG antibodies.

A support treatment of nonsteroidal anti-inflammatory drugs (NSAIDs) was initiated. Upon treatment, the fever stopped but the musculoskeletal symptoms persisted for another two weeks.

Follow-up testing from blood drawn on February 17 showed normal liver and renal function, haemoglobin, platelets, and sedimentation rates. Leucocytes had returned to normal levels (7.1 × 10^9^ cells/L).

Four weeks after the patient's blood was drawn for follow-up testing, the patient reported that symptoms had gradually improved since the beginning of March, with arthralgia and residual morning joint stiffness still present. No signs of synovitis were present upon physical examination. A presumptive diagnosis of an arbovirus infection was given.

A battery of serological tests for* Rickettsia* spp. and CHIKV was performed. No antibodies directed against rickettsial species were detected by indirect immunofluorescence. A positive CHIKV IgM enzyme-linked immunoassay (EIA) screening test [1] was obtained for the specimen collected on March 18 which was confirmed by a CHIKV plaque neutralization test (PRNT) [2] with a neutralizing antibody titer of 80. Retrospective testing of the initial specimens with the CHIKV EIA and PRNT assay revealed both IgM and neutralization antibody seroconversions between the acute specimen (Feb 7) and the convalescent specimen taken on February 21.

Amplification of the CHIKV* envelope E1* gene from both the acute and the first convalescent sample was attempted by conventional RT-PCR [3]. Successful amplification of the targeted genome (positions 10254–11170) from the acute sample resulted in the generation of a 646 bp product which was sequenced. BLAST comparison with other genotypes and phylogenic analysis indicated an identical match with a British Virgin Islands isolate [4] and showed a high sequence identity with an Asian genotype related to strains recently isolated in China and the Philippines. These results are consistent with the initial findings previously reported [4, 5] about a putative Asian origin of the strains circulating in the Caribbean.

Eight weeks after the patient first visited the doctor, the patient reported a return to normal health (lack of residual febrile syndrome, absence of hand stiffness).

## 3. Discussion

Chikungunya virus (CHIKV) is an alphavirus of the Togaviridae family and is a member of the arbovirus group. The virus is transmitted to humans mostly by the bites of the urban and periurban* Aedes aegypti* and* Aedes albopictus* mosquitoes in tropical and subtropical climates. Of importance, dengue virus is also transmitted by these arthropod vectors and coinfections with both viruses have been described [6, 7]. Following the bite of an infected mosquito and an incubation period of three to seven days, CHIKV causes a febrile illness in humans with severe polyarthralgia as a prominent symptom [8]. Despite exhibiting a number of clinical symptoms consistent with dengue infection, CHIKV is not recognized to cause the same level of severe disease as observed with dengue virus but is still responsible for significant morbidity among infected patients [9, 10].

Differential diagnosis should be carried out according to epidemiological information such as place of residence, travel history, and mosquito exposure [11]. Other zoonotic diseases such as dengue fever, Zika fever, malaria, leptospirosis, and other arboviral infections should be considered when atypical symptoms in febrile patients are observed [11]. Highly suspicious febrile cases should be investigated thoroughly with specific laboratory tests to rule out other possible infectious diseases.

The first case of CHIKV infection was reported in 1953 in Tanzania [12]. Subsequently, several waves of CHIKV epidemics (2004–2012) were observed on coastal countries of East Africa, in the islands of the Indian Ocean, India, and Italy. Over five million people were predicted to have been infected with CHIKV during these epidemics causing great morbidity and creating an economic burden in affected countries [13]. Presently, three major CHIK genotypes are circulating in the world, the west African type, the eastern, central, and southern African type (ECSA), and the Asian type [14].

CHIKV was introduced for the first time in the Americas in December 2013 on the island of Saint-Martin in the Caribbean [15]. Since then, the viral epidemic spread through local transmission cycles throughout the Caribbean as well as South, Central, and Southern North America. A viral isolate from the British Virgin Islands was collected and sequenced by the Center for Disease Control which revealed an Asian origin of the strain [4]. The Caribbean strain was nearly identical to strains circulating in the Philippines and China in 2012.

Historically, Canadian patients acquire CHIKV infections following travels to African and Asian countries where the virus is endemic and case numbers have averaged 10–15 a year [16]. As described in this report the first imported Canadian CHIKV case to be identified with travel to the Caribbean involved a resident of Québec in March 2014. More than 400 confirmed cases of CHIKV infection were documented among Canadian travellers in 2014 (Mike Drebot, unpublished observation). For 105 of the laboratory confirmed Québec cases of CHIKV diagnosed that year [16] a number of them had a confirmed travel history to the Caribbean or Central America and Table 1 shows the travel histories for representative cases. Also the most frequently reported symptoms were arthralgia, fever, and a rash (Table 1). Moreover, dengue serological testing was performed together with CHIKV testing for some patients at the request of the clinician. Seven patients with CHIKV confirmed infections were also positive for the presence of anti-dengue IgG (Table 1). The presence of IgG in the absence of IgM suggests a past infection to dengue virus. Interestingly, one patient tested in January 2015 was found to be reactive to both IgM screening tests indicating a putative CHIKV and dengue virus coinfection.

Retrospective analysis of laboratory findings for 386 Québec patients with suspected Chikungunya infection in 2014 revealed that 133 (34%) patients were positive by the EIA IgM screening test and 105 (27%) were confirmed by PRNT testing. The detection of CHIKV in acute sera by a specific RT-PCR test identified an additional 17 confirmed cases. These cases were found to be reactive by EIA but not confirmed by PRNT indicating that samples from these cases were too acute to harbor neutralizing antibodies to the virus.

According to a recent report, the number of CHIKV infection cases could rise significantly since Canada has the fourth highest number of travellers to the Caribbean (64 736 Canadian travellers between May and July 2012) [17]. In 2015 several hundred probable and confirmed Canadian cases were identified in addition to the infections documented in 2014 (Mike Drebot, unpublished observation) which reflected the continuing outbreak activity in the Americas and risk of exposure to travellers visiting endemic areas.

The envelope gene of the CHIK strain identified in this report did not exhibit the A226V substitution. This mutation was found to enable CHIKV variants to be efficiently transmitted by the Asian tiger mosquito* Ae. albopictus* which is a vector better adapted to temperate climates [18]. Based upon climate change predictive modeling programs, it was estimated that the Asian tiger mosquito population will continue to move into northern parts of the United States such as Massachusetts, New Hampshire, and Maine in the next few decades [19]. However, a recent study [20] indicates that both* Ae. albopictus* and* Ae. aegypti* populations throughout the Americas are highly competent for transmitting a variety of CHIKV genotypes. This finding is consistent with the rapid spread of the virus currently observed and the accompanying outbreak of CHIKV disease throughout the Caribbean. Seven months following the first case in America, eleven locally acquired CHIKV infection cases were reported in Florida in June 2014 [21]. Presently, the risk of local transmission of CHIKV in Canada is quite low. The proper ecological and climate conditions required to foster the establishment of CHIKV in Canada are not found at this time. However, the predicted climate change over the next decades could affect the probability of emergence of competent mosquito vectors and consequently the establishment of CHIKV in southern parts of Canada [22].

Several thousands of people are travelling to the islands of the Caribbean each year and consequently are at risk of being infected by dengue, CHIKV, or the recently introduced Zika virus (ZIKAV). Similarly to the dengue virus, ZIKAV is a flavivirus which is found in some African and Asian countries. ZIKAV was detected for the first time in Brazil in March 2015 and has spread rapidly to Central America and the Caribbean [23]. Prompt serological testing for these virus infections is recommended for febrile patients with polyarthralgia who travelled to countries where autochthonous arboviral transmission is ongoing. In addition, the development of surveillance programs able to detect and measure the CHIKV progression is a prerequisite to adequately prepare and guide health authorities to make appropriate interventions in the case of a continental emergence and establishment of CHIKV in Canada.

## Figures and Tables

**Table 1:** Canadian imported Chikungunya infection cases with documented travel history and clinical symptoms.

Collection date	Age	Gender	Test results					Country visited	Symptoms
			Chikungunya			Dengue			
			EIA IgM	PRNT	RT-PCR	EIA IgM	EIA IgG		
March 18, 2014	57	M	POS	1/80	NT	NEG	NEG	Martinique	Fever, rash, arthralgia
May 25, 2014	59	M	POS	>1/80	NT	NEG	POS	Haiti	Headache, high hepatic enzyme
June 10, 2014	71	F	POS	>1/80	NT	NEG	POS	Haiti	NA
July 10, 2014	20	F	POS	>1/80	NT	NEG	NEG	Haiti	NA
July 15, 2014	55	M	POS	NEG	POS	NEG	NEG	Guadeloupe	Fever
July 22, 2014	41	F	POS	1/20	POS	NEG	NEG	Haiti	Fever
July 29, 2014	59	F	POS	1/40	NT	NT	NT	Haiti	Headache, rash, arthralgia, myalgia
July 29, 2014	31	F	POS	1/80	NT	NEG	NEG	Dominican Republic	NA
August 06, 2014	53	M	POS	1/80	NT	NEG	POS	Haiti	Fever, arthralgia
August 01, 2014	51	F	POS	1/80	NT	NEG	POS	Haiti	Arthralgia, myalgia, asthenia
August 18, 2014	24	F	POS	1/80	NT	NT	NT	Haiti	NA
August 28, 2014	49	M	EQV	1/20	NT	NEG	NEG	Dominican Republic	NA
September 19, 2014	74	M	POS	1/160	NT	NT	NT	Dominican Republic	Arthralgia
September 23, 2014	43	F	EQV	NT	POS	NEG	POS	El Salvador	Fever, rash, arthralgia
September 26, 2014	60	F	POS	1/40	NT	NT	NT	Dominican Republic	NA
October 06, 2014	34	F	POS	>1/80	NT	NT	NT	Grenada	Arthralgia
November 04, 2014	49	F	POS	1/40	NT	NEG	NEG	Grenada	Polyarthralgia
November 10, 2014	57	F	POS	1/20	NT	NT	NT	Jamaica	NA
November 12, 2014	48	F	POS	NEG	POS	NEG	NEG	Jamaica	Fever, rash, arthralgia
November 21, 2014	38	F	POS	>1/160	NT	NEG	POS	Puerto Rico	NA
January 15, 2015	44	M	POS	NEG	POS	POS	POS	Guatemala	Arthralgia, rash

EQV, equivocal; NA, not available; NEG, negative; NT, not tested; POS, positive.
